# Book Review

**Published:** 1901-03

**Authors:** 


**Year-Book of the Scientific and Learned Societies of Great Britain and Ireland.** Seventeenth Annual Issue. London: Charles Griffin & Company, Limited. 1900.—A well-arranged and indexed summary of work done and papers read in various learned societies during the past year, which is of great value for purposes of reference; the care and completeness with which this has been compiled deserve high commendation.

**Illustrated Lectures on Nursing and Hygiene.** By R. Lawton Roberts, M.D. Third Edition. Pp. xxv., 223. London: H. K. Lewis. 1900.—The contents of this book are mainly arranged on the plan of the syllabus of the advanced nursing course of the St. John Ambulance Association. The preface to this edition states that "a very cursory examination of the reports of the St. John Ambulance Brigade shows that not only is a vast amount of useful public work done at home and, as in the present war, abroad by the Brigade, but that the Nursing Divisions, acting outside the Brigade as local nursing societies in their respective districts, carry out unostentatiously a great benevolent, self-denying, and self-imposed duty of ministering to the sick pocr in their own homes." These lectures are likely to be of great utility to all such persons, and an appendix on filters, tuberculous disease, and disinfection gives the most recent information on these topics.

**The Johns Hopkins Hospital Reports.** Vol. VII., No. 4.—*The Origin, Growth and Fate of the Corpus Luteum as Observed in the Ovary of the Pig and Man.* By J. G. Clark, M.D. Baltimore: The Johns Hopkins Press. 1898.—This pamphlet, which embodies a most important contribution to the literature of the subject of the function and development of the corpus luteum, will prove instructive to the physiologist and histologist. It must be read in its entirety, since the space of a review can give but the baldest outline of the arguments deduced from the author's observations, unless the observations themselves are described in detail. It is sufficient to say that after describing the main arguments used by previous workers as to the origin and development of the corpus luteum—namely (1) The view of Von Baer and others that the lutein cells are developed from the connective tissue cells of the theca interna; (2) The view of Bischoff that the chief factors in the origin of the lutein cells are those of the membrana granulosa; and (3) The view of Patterson and Henle that the chief factor is the coagulation of blood in the Graafian follicle,—the author, working on lines independent of theirs, and without preconceived ideas, considers he has proved that the view of Von Baer is correct and that the course of origin of the lutein cells is from the connective tissue cells of the theca interna, which become specalised. He considers the function of the corpus luteum to be that of the preservation of the circulation in the ovarian stroma, and so of the function of ovulation, while the cessation of ovulation is induced, not by the disappearance of follicles, but through a densification of the ovarian stroma and destruction of the peripheral circulation, which prevents the development of the follicles. The course of investigation, and the histological methods described, are most complete and interesting. Some capital engravings of photo-micrographs which are added elucidate the text considerably.

**The Student's Medical Dictionary.** By George M. Gould, M.D. Eleventh Edition. London: H. K. Lewis. 1900.—The indebtedness of the profession to Dr. Gould grows year by year. The new edition of this book shows a marked improvement over its forerunners, although we are sorry to see that attention has not been given to some slight inaccuracies and omissions to which we referred in our notice of the tenth edition. Not only to the student, for whom it is particularly intended, but also to the practitioner, its value is great, and not easily stated in the words of a necessarily short review. The advancing nomenclature of medicine requires that such a volume should be at the easy command of everyone engaged in any of the multifarious phases of professional work, and we recommend all those so occupied to provide themselves with this book, which not only has a wealth of informing definitions, but is enriched by tables of various kinds admirably prepared by Dr. Gould. This edition has a large number of pictures which the previous ones lacked. We were astonished that we had to look in vain for Achondroplasia.

**Plague. How to Recognise, Prevent, and Treat Plague.** By James Cantlie, M.B. Pp. 66. London: Cassell & Company, Limited. 1900.—"The appearance of plague in Glasgow, in August, 1900, has brought the subject home to the people in this country, so keenly, that it behoves every medical man to know something of a disease which develops so insidiously and encroaches so stealthily that it may escape recognition, until a serious hold on the community may be obtained before its presence is recognised." This timely pamphlet will enable any medical man to diagnose an unfamiliar disease. "A pneumonia which carries off two or three persons in a house, typhoid or typhus fever to which a query is attachable, convulsions in children of undetermined origin and with a rapidly fatal issue, glandular fever in children, and many other conditions and symptoms, ought to suggest to the doctor's mind, if plague is in the country, that this untoward infection may play a part in the disease roll."

